# Title, Table of Contents and Acknowledgements

**DOI:** 10.1080/26410397.2019.1705115

**Published:** 2019-12-30


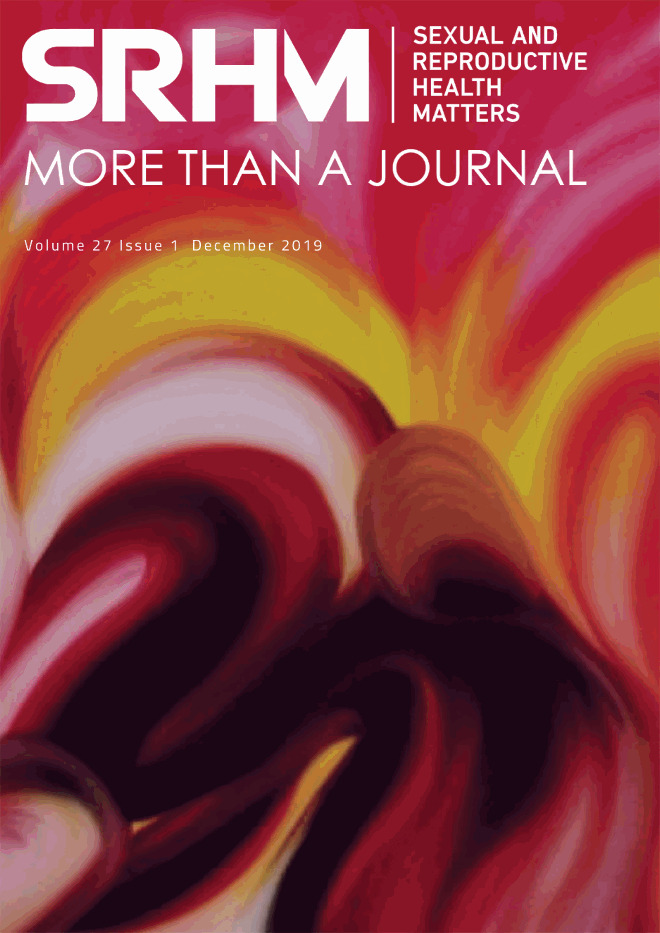


**Editorial**

1 *Jane Cottingham, Eszter Kismödi, Julia Hussein*

*Sexual* and Reproductive Health Matters – What's in a name?

**Commentaries**

4 *Joanna N Erdman*

The gender injustice of abortion laws

9 *Caitlin R Williams, Benjamin Mason Meier*

Ending the abuse: the human rights implications of obstetric violence and the promise of rights-based policy to realise respectful maternity care

12 *Jennifer Thomson, Fran Amery, Melanie Channon, Mahesh Puri*

What's missing in MHM? Moving beyond hygiene in menstrual hygiene management

**Review articles**

16 *Cynthia Khamala Wangamati, Gladys Yegon, Johanne Sundby, Ruth Jane Prince*

Sexualised violence against children: a review of laws and policies in Kenya

29 *Sofia Gruskin, Vithika Yadav, Antón Castellanos-Usigli, Gvantsa Khizanishvili, Eszter Kismödi*

Sexual health, sexual rights and sexual pleasure: meaningfully engaging the perfect triangle

41 *Verónica Undurraga*

Criminalisation under scrutiny: how constitutional courts are changing their narrative by using public health evidence in abortion cases

52 *Gianna Robbers, Joshua P Vogel, Glen Mola, John Bolgna, Caroline SE Homer*

Maternal and newborn health indicators in Papua New Guinea – 2008-2018

69 *Thuy Trinh, Alexis F Leal, Maeve B Mello, Melanie M Taylor, Roxanne Barrow, Teodora E Wi, Mary L Kamb*

Syphilis management in pregnancy: a review of guideline recommendations from countries around the world

**Research articles**

83 *Charlotte Kühlbrandt*

Confronting racism in family planning: a critical ethnography of Roma health mediation

93 *Susan B Schaffnit, Mark Urassa, David W Lawson*

“Child marriage” in context: exploring local attitudes towards early marriage in rural Tanzania

106 *Sara E Casey, Victoria J Steven, Julianne Deitch, Erin Files Dumas, Meghan C Gallagher, Stephanie Martinez, Catherine N Morris, Raoza Vololona Rafanoharana, Erin Wheeler*

“You must first save her life”: community perceptions towards induced abortion and post-abortion care in North and South Kivu, Democratic Republic of the Congo

118 *Courtney Kerestes, Kelsey Sheets, Colleen K Stockdale, Abbey J Hardy-Fairbanks*

Prevalence, attitudes and knowledge of misoprostol for self-induction of abortion in women presenting for abortion at Midwestern reproductive health clinics

126 *Diana Suk-Chin Law, Chai-Eng Tan, Seng-Fah Tong*

Influences on the decision to use contraception among Sarawakian women with diabetes: a qualitative exploration

136 *Diane Duclos, Francesca L Cavallaro, Tidiane Ndoye, Sylvain L Faye, Issakha Diallo, Caroline A Lynch, Mareme Diallo, Adama Faye, Loveday Penn-Kekana*

Critical insights on the demographic concept of “birth spacing”: locating *Nef* in family well-being, bodies, and relationships in Senegal

146 *Goleen Samari*

Women's empowerment in Egypt: the reliability of a complex construct

160 *Cynthia Beavin, Deborah L Billings, Susana Chávez*

Activist framing of abortion and use for policy change in Peru

168 *Hiroaki Matsuura*

Exploring the association between the constitutional right to health and reproductive health outcomes in 157 countries

181 *Maureen Murphy, Jeffrey B Bingenheimer, Junior Ovince, Mary Ellsberg, Manuel Contreras-Urbina*

The effects of conflict and displacement on violence against adolescent girls in South Sudan: the case of adolescent girls in the Protection of Civilians Site in Juba

192 *Camilla Palm, Birgitta Essén, Sara Johnsdotter*

Sexual health counselling targeting girls and young women with female genital cutting in Sweden: mind-body dualism affecting social and health care professionals’ perspectives

203 *Heidi Moseson, Ramatou Ouedraogo, Soukeyna Diallo, Amy Sakho*

Infanticide in Senegal: results from an exploratory mixed-methods study

215 *Catriona A Towriss, Tamsin K Phillips, Kirsty Brittain, Allison Zerbe, Elaine J Abrams, Landon Myer*

The injection or the injection? Restricted contraceptive choices among women living with HIV

228 *Janet Perkins, Ahmed Ehsanur Rahman, Shema Mhajabin, Abu Bakkar Siddique, Tapas Mazumder, Mohammad Rifat Haider, Shams El Arifeen*

Humanised childbirth: the status of emotional support of women in rural Bangladesh

248 *Dominick Shattuck, Sharada P Wasti, Naramaya Limbu*^*c*^ *Nokafu Sandra Chipanta, Christina Riley*

Men on the move and the wives left behind: the impact of migration on family planning in Nepal

262 *Ruth Nara, Amanda Banura, Angel M Foster*

Exploring Congolese refugees’ experiences with abortion care in Uganda: a multi-methods qualitative study

272 *Hartley Feld, Verónica Rojas, Ana Maria Linares*

“We keep quiet”: exploring the context of pregnancy intention in a low-resource community in Ecuador

287 *Gilbert Gravino, Liza Caruana-Finkel*

Abortion and methods of reproductive planning: the views of Malta's medical doctor cohort

**Perspectives**

304 *Amit Sengupta*

Maternal health in underserved tribal India

307 *Ernst Patrick Graamans, Eefje Smet, Steven ten Have*

Legislation against girl circumcision: a cultural psychological understanding of prohibition

310 *Jean Jose (Jimmy) Nzau, Benediction Mbaikar Denemadjbe, Erin Files Dumas, Mariela A Rodriguez*

Catalysing change for reproductive health in Chad through a multi-stakeholder coalition

313 *Antón Castellanos-Usigli, Doortje Braeken-van Schaik*

The Pleasuremeter: exploring the links between sexual health, sexual rights and sexual pleasure in sexual history-taking, SRHR counselling and education

316 *Carly A Comins, Sheree R Schwartz, Katherine Young, Sharmistha Mishra, Vijayanand Guddera, Mfezi Mcingana, Deliwe R Phetlhu, Harry Hausler, Stefan Baral*

Contextualising the lived experience of sex workers living with HIV in South Africa: a call for a human-centred response to sexual and reproductive health and rights

**Collection: Accelerating accountability for SRHR**

319 *Gita Sen, Eszter Kismödi, Anneka Knutsson*

Moving the ICPD agenda forward: challenging the backlash

323 *Natalia Kanem*

The battle for sexual and reproductive health and rights for all

326 *Rebecca Brown, Eszter Kismödi, Rajat Khosla, S Malla, Lucy Asuagbor, Ximena Andión-Ibanez, Sofia Gruskin*

A sexual and reproductive health and rights journey: from Cairo to the present

329 *Rajat Khosla, Avni Amin, Pascale Allotey, Carmen Barroso, Asha George, Anita Hardon, Ian Askew*

“Righting the wrongs”: addressing human rights and gender equality through research since Cairo

333 *Susana T Fried, Aziza Ahmed, Luisa Cabal*

Tensions and exclusions: the knotty policy encounter between sexual and reproductive health and rights and HIV

336 *Venkatraman Chandra-Mouli, Marina Plesons, Arup Barua, Anshu Mohan, Meheret Melles-Brewer, Danielle Engel*

Adolescent sexual and reproductive health and rights: a stock-taking and call-to-action on the 25^th^ anniversary of the International Conference on Population and Development

340 *Tom Shakespeare, Shaffa Hameed, Lizzie Kiama*

Actions, not words: progress since ICPD on disability and SRHR

343 *Shirin Heidari, Monica A Onyango, Sarah Chynoweth*

Sexual and reproductive health and rights in humanitarian crises at ICPD25+ and beyond: consolidating gains to ensure access to services for all

346 *Claudia Garcia-Moreno, Avni Amin*

Violence against women: where are we 25 years after ICPD and where do we need to go?

349 *Priya Nanda, Sahil Tandon*

“The Times They Are A-Changin”: using technology to enable ASRHR 25 years post-ICPD

**Editor-in-Chief:** Julia Hussein

**Chief Executive:** Eszter Kismödi

**Managing Editor:** Pete Chapman, Sarah Pugh

**Monitoring Editor:** Pathika Martin

**Communications Manager:** Jessica MacKinnon

**Communications Officer:** Alexane Bremshey

**Finance Manager:** Elisabeta Pashaj, Lance Stewart

**Operations Manager:** Edna Epelu

**Associate Editors:** Laura Ferguson, Nambusi Kyegombe, Emma Pitchforth, Mindy Jane Roseman, Nina Sun, Joyce Wamoyi

Peer reviewers:

Ayse Nurdagul Akin, Ababe Alemu Anshebo, Samuel Kojo Antobam, Sarah Ashraf, Mary Rose Bagita, Elizabeth Bartelt, Hyam Bashour, Jacqueline Bell, Eran Bendavid, Maria Berghs, Isha Bhallamudi, Deborah Billings, Fiona Bloomer, Pablo Shiladitya Boze, Naomi Braine, Shyam Sundar Budhathoki, Megan Casebolt, Sarah Casey, John B Casterline, Dennis Fuh Chenwi, Manuela Colombini, Rasha Dabash, Sapna Desai, Nayana Dhavan, Farah Diaz-Tello, Andrea Dibben, Kwamena Sekyi Dickson, Jatinder Dillon, Deborah Diniz, Sandra Downing, Diane Duclos, Meghan Eagen-Torkko, Gillian Einstein, Lisa Eklund, Hala Eldamanhoury, Khalifa Elmusharaf, Olivia Engle, Dabney P Evans, Nkoli Nwakego Ezumah, Bridget Ferguson, Laura Ferguson, Jessica Fields, Rebecca Fish, Melissa Garcia, Sandra Garcia, Camila Gianella, Margaret Giorgio, Nancy E Glass, Ellen Gruenbaum, Bridget Haire, Siobán D Harlow, Kahabi Isangula, Smarajit Jana, Ruvani Jayaweera, Saira Parveen Jolly, Rachel Jones, Hari Bahadur Karki, Angela Kelly-Hanku, Eszter Kismödi, Vesile Koçak, Sandra Krause, Nambusi Kyegombe, Nistha Lamba, Laura Laski, Genesis Luigi, Therese McGinn, Kazuyo Machiyama, Monica Magadi, Rupsa Malik, Rozliza Manaf, Chi Mgbako, Celine Miani, Elizabeth A Mosley, Moses Ndegwa Mutiga, Ndeye Fatou Ndiaye, Peter Ngatia Nguura, Michelle Oberman, Jenny O'Donnell, Oduro Oppong-Nkrumah, Camilla Palm, Anne Philpott, Melanie Pleaner, Rodolfo Gomez Ponce de Leon, Bill Powell, Latika Maskey Pradhan, Manas Ranjan Pradhan, Sarah Pugh, Martha Rac, Akila Radhakrishnan, Emma Radovich, Ana Maria Ramirez, TK Sundari Ravindran, Mohammad Rifat Haider, Michele Rivkin-Fish, Kathryn W Roberts, Mindy Jane Roseman, Sharmila Rudrappa, Kabiru Salami, Pikee Saxena, Frederic Seghers, Antonio Serafim, ASM Shahabuddin, Mridula Shankar, Atef el-Shitany, Suzanne Sicchia, Kavita Singh, Chander Skekhar, Michael Stambolis-Ruhstorfer, Karin Stanzel, Nina Sun, Johanne Sundby, Nicolas Syrett, Jamie Vernaelde, Katherine Wade, Marius Wamsiedel, Felicia Yeung

**Cover image:** Reshma Valliappan aka Val Resh, Untitled 19, 2008, from oil painting collection ‘The Awakening’. “*The Awakening is a series of art work that begun in 2008 and continued till late 2011. It marked my journey of leaving my medications and moving away from conventional understanding of psychosis and schizophrenia.*”

Translation:

Françoise de Luca-Lacoste translated abstracts from English to French and Lisette Silva translated abstracts from English into Spanish.

**Copyright © 2019**

**Sexual and Reproductive Health Matters**. This is an Open Access journal distributed under the terms of the Creative Commons Attribution License (http://creativecommons.org/licenses/ by/4.0/), which allows for sharing and adapting the work for any purpose, even commercially, provided appropriate credit is given with a link to the originally published item, a reference to the author(s) and links to their homepages, reference to the license under which the article is published and a link to this, as well as an indication of any changes that have been made to the original.

ISSN (Online) 2641-0397

**SRHM in translation online**

Selected papers from the SRHM journal are published in Arabic, Chinese, French, Hindi, Portuguese, Russian and Spanish. Go to: http://www.srhm.org/our-journals/

**Funding**

SRHM’s work in 2019 has been supported by the Open Society Foundations, the United Nations Population Fund (UNFPA) and the Women's Refugee Commission.

Authors are responsible for the content of their articles which do not necessarily reflect positions or policies of the funders.

www.srhm.org / www.srhmjournal.org

Twitter @SRHMJournal

Facebook @SRHMJournal

